# A descriptive study of high resolution total colonic intracavitary manometry and colonic transit test in the diagnostic efficacy of functional constipation in Chinese patients

**DOI:** 10.1186/s12876-022-02240-x

**Published:** 2022-04-09

**Authors:** Dan Wang, Zhao Zhang, Mingsen Li, Tingting Liu, Chao Chen, Jiying Cong, Chenmeng Jiao, Yuwei Li

**Affiliations:** 1 Department of Pathology, Tianjin Medical University General Hospital, Tianjin, 300052 People’s Republic of China; 2 Department of Colorectal Surgery, Tianjin Union Medical Center, Tianjin, 300121 People’s Republic of China

**Keywords:** Functional constipation, Colonic transit test, Colonic intracavitary manometry, Diagnosis, Efficacy

## Abstract

**Background:**

This study was to observe the diagnostic efficacy of high resolution total colonic intracavitary manometry (HRCM) vs colonic transit test (CTT) in the assessment of functional constipation (FC) in Chinese patients.

**Methods:**

Seventy-nine cases of patients with severe FC who were admitted and received colon resection between July 2016 and July 2019 at the Tianjin Union Medical Center were retrospectively reviewed. Before operation, all patients received CTT at outpatient service, followed by HRCM at ward. The resected tissues were subject to histological observation, which was used to determine the diagnostic efficacy of HRCM vs CTT.

**Results:**

The accuracy of CTT for the FC diagnosis was 69.6% (55/79), and the false negative ratio was 30.4%. The accuracy of HRCM for the FC diagnosis was 81.0% (64/79), and the false negative ratio was 19.0% (15/79). Twelve patients showed normal characteristics after CTT but abnormal after HRCM. In contrast, only 4 showed normal after HRCM but abnormal after CTT. In addition, among the 79 patients 12 were detected normal by both CTT and HRCM.

**Conclusion:**

HRCM can be more suitable to assess FC compared with CTT, while CTT is still indispensable for HRCM to diagnose FC.

## Background

In China the prevalence of constipation was 8.2% in general population, which was particularly higher in the elderly and pediatric populations (18.1% and 18.8%, respectively) [1]. Slow-transit constipation (STC) is the most common type of constipation. Functional constipation (FC), mainly including STC and caused by various factors except intestinal or systemic organic pathological ones, is characterized by reduced stool frequency, prolonged stool interval, less fecal volume and hard quality, and difficulty in defecation. The etiology of constipation is still unclear, and currently there are no related specific drugs in clinics. For patients without response to drugs, surgical treatment is needed. Surgery is mainly to resect the colons without peristaltic function, but before the operation it is necessary to exclude the cases of small bowel dysfunction and gastric emptying disorder. However, there are still no specific methods for the examination of colonic functions. The present guideline just recommends the colonic transit test (CTT) to assess the general colonic peristalsis functions based on the imaging features, which can neither be used to determine the exact part of colons with poor peristalsis function nor to assess the extent of the peristaltic function attenuation. Therefore, the current guideline has limited value for instructing such clinical surgeries.

During surgery, to decide whether or not to retain the ileocecal parts and ascending colons, it is necessary to assess the functions of cecum and ascending colons, which is difficult to be evaluated through CTT. The colonic manometry can accurately determine the peristaltic pressure in all parts of the colons and the trend of colonic peristalsis to instruct the choice of appropriate clinical surgical means. Colonic manometry has been used in the diagnosis of chronic constipation [2]. For example, Dranove et al. used colonic manometry to evaluate the aspects of colonic functions in children with refractory constipation [3]. Currently, the commonly used facility emergingly involved non-high-resolution (low-resolution) catheters [4], and low-resolution colonic manometry has some disadvantages, e.g., it may bring about gross misinterpretation of the frequency, morphology and polarity of the colonic propagating sequences, key for the movement of colonic content and defecation [5].

Recently, high-resolution colonic manometry (HRCM), with more closely spaced sensors, has been rapidly developing for more effectively evaluating the colonic peristaltic contractions of the entire human colons, such as colon motility, manometry, operation, and colonic and anal motor activity [6–13], in the diagnosis and classification of constipation. For example, Li et al. used HRCM to effectively determine the colonic motility patterns and morphological changes in the colonic walls of patients (151 cases) with constipation to diagnose and classify the types of constipation as well as accurately identify the diseased colonic segments in order to optimize the appropriate treatment [6]. Sintusek et al. applied HRCM to assess the colonic motor activity in children with treatment-unresponsive STC [7].

HRCM can provide more detailed information about the constipation. However, CTT, with limited beneficial information, is still recommended in the guidelines for the FC treatment. It is important to assess if HRCM can be superior to and substitute CTT in the diagnosis of constipation. Until now, however, there are no such reports yet. In this study, we reviewed the clinical information of patients with FC who underwent the CTT and whole HRCM in sequence before colonectomy so as to compare the efficacy of these two methods in diagnosis of FC.

## Methods

### Patients

Patients with FC who were admitted to the Tianjin Union Medical Center (Tianjin, China) during July 2016 and July 2019 were retrospectively reviewed. Patients met the Roman IV Functional Constipation Criteria and were pathologically confirmed to have FC. Patients, who did not meet the Roman IV criteria, just had hirschsprung, had no pathological results, or had mental illness, were excluded. This study was approved by the Ethics Committee of the Tianjin Union Medical Center, and the patients’ anonymity were strictly maintained. Written informed consent was obtained from all participants.

### HRCM

Referred to Li et al.’s method [6]. Solar GI high HRCM machine (Medical Measurement System, Holland) was applied to record the colorectal motility. Briefly, the catheter was first kept horizontally at the level of patient’ gut to make the pressure zero-balance and was then moved vertically to check the registration of the transducers. Under the guiding of colonoscope, a solid-state catheter (2.0 cm long and 0.45 cm in diameter) with 36 channels was placed in different segments of colons (rectum, and sigmoid and descending colons), 5 cm apart from each other. The minimum pressure was obtained by calculating all the channel pressures minus their offset pressures. HRCM lasted for over 24 h, and the amplitude, intensity and trends in the whole colonic peristaltic contractions were monitored and recorded in the states of rest, after meals, during sleep and wakefulness. Manometric data were analyzed and displayed as pressure topography using the Medical Measurement Systems software. Low amplitude peristaltic contraction (LAPC), which was defined as contraction amplitude < 75 mmHg but > 5 mmHg [6], represented HRCM positive (abnormal) result, including myogenic, partial myogenic, neurogenic, partial neurogenic, and mixed (combination of myogenic with neurogenic) constipation. [6].

### CTT

CTT was carried out before the operation according to the routine protocols. Briefly, patients were orally administered labelled drugs and were measured their movement and distribution by X-ray examination. CTT results included pathologic and mixed (mixture of pathologic and functional outlet obstruction) constipation.

### Diagnosis of constipation

Constipation was diagnosed according to the Rome criteria IV [6], including: (1) the presence, in the preceding 6 months, of at least 2 of the following: (i) straining at the time of > 1/4 defecations, (ii) a sensation of incomplete evacuation at the time of > 1/4 defecations, (iii) a sensation of anorectal obstruction/blockage at the time of > 1/4 defecations, and (iv) manual maneuvers to facilitate > 1/4 defecations (e.g., digital evacuation, or need for pelvic floor support), (2) no loose stool without the use of laxative, and (3) inadequate evidence for the diagnosis of irritable bowel syndrome. Constipation was further confirmed by histological observation through hematoxylin and eosin (HE) staining on the resected colonic tissues according to our previous report [6], which was used to validate the CTT and HRCM examination results.

## Results

Seventy-nine cases of subjects with STC, who received CTT and HRCM in sequence and then underwent surgical resection of the colon tissues, were reviewed, including 15 males and 64 females. The median age was 58 (range 26–77) years. The median Wexner score upon admission was 12 (range 2–22). All the resected colon tissue samples were paraffin-embedded and subject to the histological observation by HE staining, indicating vacuolar degeneration of nerve plexuses and muscularis propria and fibrosis in the outer layers (typical HE staining of the resected colon samples was shown in Figs. 3C, 4C and 5C), which confirmed the diagnosis of constipation in all the 79 samples.

**Fig. 1:**
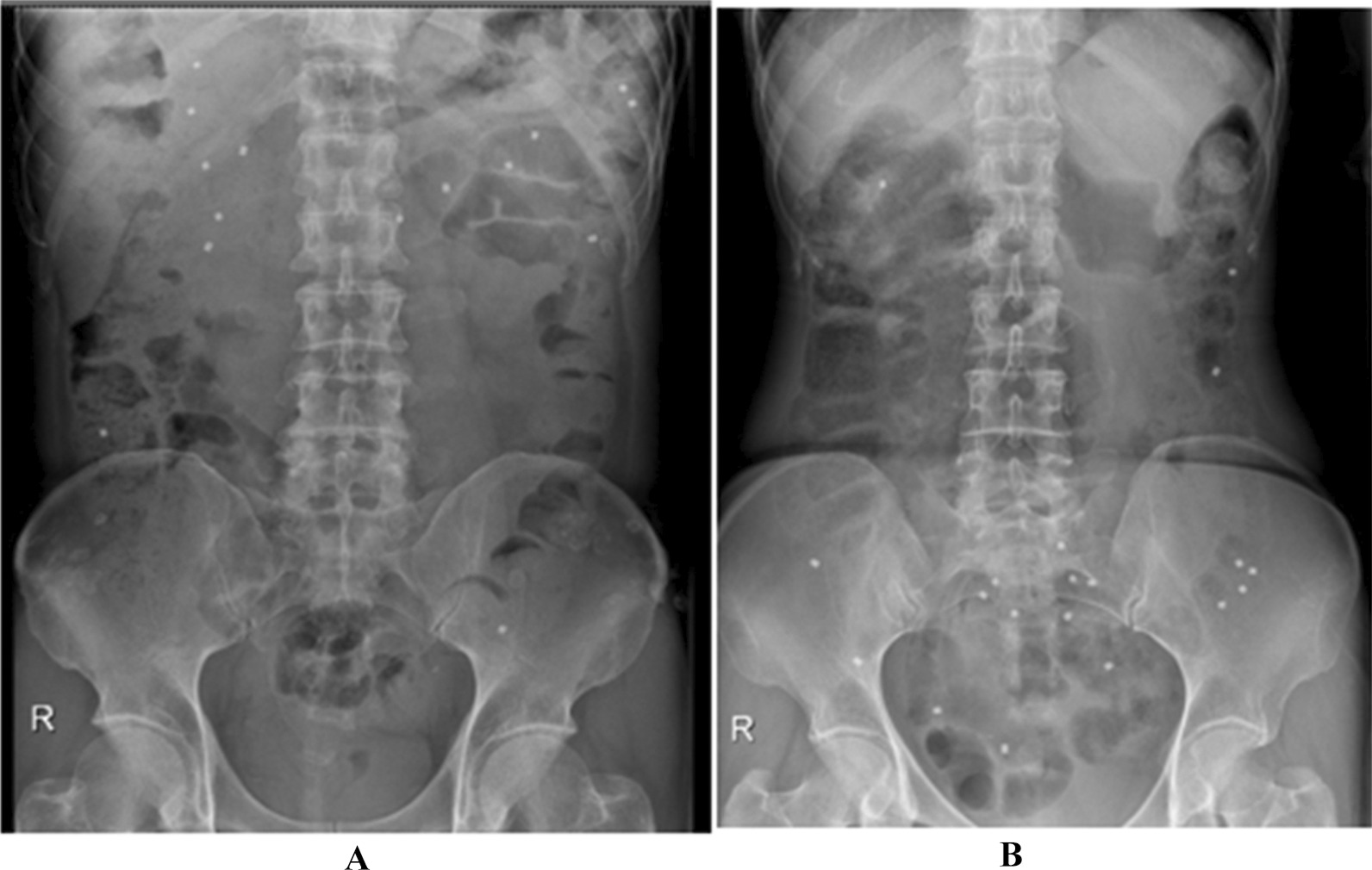
Representative CTT images in FC patients (Case 3 and 6). **A** Pathologic FC in Case 3 by CTT. **B** Mixed (combination of pathologic and defecation disorder type) FC in Case 6 by CTT

**Fig. 2:**
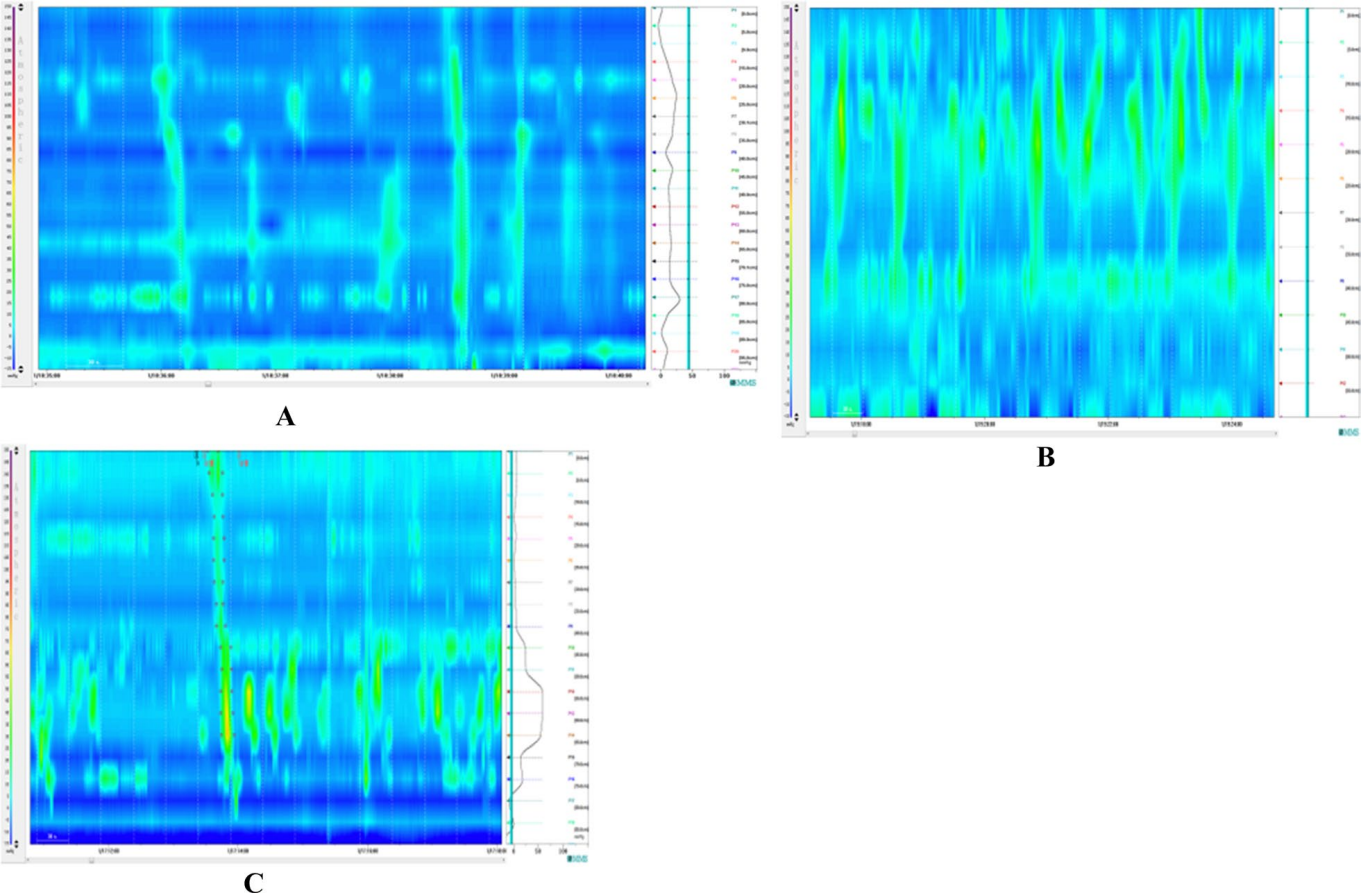
Representative HRCM positive (with LAPC waves) images in FC patients (Case 3 and 25). **A** myogenic FC in Case 3 by HRCM. **B** Neurogenic FC in Case 25 by HRCM. **C** Mixed (myogenic and neurogenic) FC in Case 51 by HRCM

The detailed information of the patients was shown in Table 1. Representative CTT images in FC patients were shown in Fig. 1, including pathogenic FC in Case 3 (Fig. 1A), and mixed FC in Case 6 (Fig. 1B). For CTT examination, 24 (#15, 21, 23, 38, 48, 52, 54, 61–73, 76–79) of the 79 cases, which were confirmed to have FC by histological observation, were normal after CTT. The accuracy of CTT for STC diagnosis was 69.6% (55/79), and the false negative ratio was 30.4%. Figure 2 showed representative HRCM images in FC patients, including myogenic FC in Case 3 (Fig. 2A), neurogenic FC in Case 25 (Fig. 2B), and mixed FC in Case 51 (Fig. 2C). In contrast, 15 (#15, 21, 33, 64–66, 69–72, 74–76, 78, 79) of 79 were normal after HRCM. The accuracy of HRCM for STC diagnosis was 81.0% (64/79), and the false negative ratio was 19.0%.

**Table 1:** CTT and HRCM observation of constipation patients

Patient	Sex	Age	Wexner	CTT (type)	HRCM (type)	Surgical treatment
1	Female	60–65	13	Pathologic	Myogenic	Laparoscopic subtotal colectomy, and lateral side-to-side anastomosis of ascending colon and rectum
2	Female	50–55	13	Pathologic	Myogenic	Laparoscopic subtotal colectomy, lateral side-to-side anastomosis of ascending colon and rectum, and terminal ileostomy
3	Male	50–55	11	Pathologic	Myogenic	Laparoscopic subtotal colectomy, lateral side-to-side anastomosis of ascending colon and rectum, terminal ileostomy, and colonoscopic hemostasis of anastomotic bleeding
4	Female	50–55	12	Pathologic	Myogenic	Laparoscopic subtotal colectomy, lateral side-to-side anastomosis of ascending colon and rectum
5	Female	65–70	7	Pathologic	Myogenic	Laparoscopic total colectomy, ileoproctostomy, and terminal ileostomy
6	Female	45–50	12	Mixed^1^	Myogenic	Laparoscopic subtotal colectomy, lateral side-to-side anastomosis of ascending colon and rectum
7	Male	75–80	8	Pathologic	Myogenic	Laparoscopic subtotal colectomy, lateral side-to-side anastomosis of ascending colon and rectum, and terminal ileostomy
8	Female	55–60	14	Mixed^1^	Partial myogenic	Laparoscopic subtotal colectomy, lateral side-to-side anastomosis of ascending colon and rectum
9	Female	55–60	9	Pathologic	Mixed^2^	Laparoscopic total colectomy, adhesiolysis, appendectomy and ileoproctostomy
10	Male	60–65	12	Pathologic	Myogenic	Laparoscopic partial colectomy, enterolysis, appendectomy, peritoneal drainage, and colostomy
11	Female	65–70	13	Mixed^1^	Mixed^2^	Laparoscopic total colectomy, enterolysis, lateral side-to-side anastomosis of ascending colon and rectum, terminal ileostomy
12	Male	65–70	10	Pathologic	Mixed^2^	Laparoscopic total colectomy
13	Female	65–70	9	Pathologic	Myogenic	Laparoscopic total colectomy, enterolysis, and permanent terminal ileostomy
14	Female	40–45	12	Mixed^1^	Myogenic	Laparoscopic total colectomy, enterolysis, peritoneal drainage, and ileoproctostomy
15	Female	40–45	14	Normal	Normal	Laparoscopic total colectomy, ileoproctostomy
16	Female	60–65	13	Mixed^1^	Myogenic	Laparoscopic total colectomy, enterolysis, peritoneal drainage, and permanent terminal ileostomy
17	Female	50–55	17	Mixed^1^	Myogenic	Laparoscopic total colectomy, appendectomy, enterolysis, peritoneal drainage, and lateral side-to-side anastomosis of ascending colon and rectum
18	Male	75–80	11	Mixed^1^	Myogenic	Laparoscopic subtotal colectomy, lateral side-to-side anastomosis of ascending colon and rectum, and terminal ileostomy
19	Female	50–55	13	Pathologic	Partial neurogenic	Laparoscopic total colectomy, ileoproctostomy, ileostomy, and complex adhesiolysis
20	Male	60–65	13	Pathologic	Partial myogenic	Laparoscopic subtotal colectomy, anastomosis of ascending colon and rectum, and complex adhesiolysis
21	Female	65–70	10	Normal	Normal	Laparoscopic total colectomy
22	Female	35–40	12	Pathologic	Myogenic	Laparoscopic total colectomy, and prophylactic ileostomy
23	Male	65–70	14	Normal	Myogenic	Laparoscopic subtotal colectomy, and lateral side-to-side anastomosis of ascending colon and rectum
24	Male	55–60	10	Mixed^1^	Myogenic	Laparoscopic total colectomy, and ileoproctostomy
25	Female	25–30	8	Mixed^1^	Neurogenic	Laparoscopic total colectomy, and ileoproctostomy
26	Female	55–60	12	Pathologic	Myogenic	Laparoscopic subtotal colectomy, and lateral side-to-side anastomosis of ascending colon and rectum
27	Female	70–75	20	Pathologic	Mild myogenic	Laparoscopic total colectomy, enterolysis, ileoproctostomy, peritoneal drainage, and ileostomy
28	Female	65–70	17	Mixed^1^	Mixed^2^	Laparoscopic total colectomy, enterolysis, preventive ileostomy, and peritoneal drainage
29	Female	70–75	12	Pathologic	Myogenic	Laparoscopic total colectomy, ileoproctostomy, ileostomy, complex enterolysis, and repairment of incisional hernia
30	Female	60–65	17	Pathologic	Myogenic	Laparoscopic subtotal colectomy, and lateral side-to-side anastomosis of ascending colon and rectum
31	Female	65–70	8	Pathologic	Partial myogenic	Laparoscopic total colectomy, ileoproctostomy, and peritoneal drainage
32	Female	70–75	22	Pathologic	Mild myogenic	Laparoscopic subtotal colectomy, and lateral side-to-side anastomosis of ascending colon and rectum
33	Female	75–80	2	Mixed^1^	Normal	Laparoscopic total mesorectal excision, and preventive ileostomy
34	Female	70–75	11	Pathologic	Myogenic	Laparoscopic total colectom, and ileoproctostomy
35	Male	60–65	8	Mixed^1^	Myogenic	Laparoscopic subtotal colectomy, lateral side-to-side anastomosis of ascending colon and rectum, and ileostomy
36	Female	30–35	11	Mixed^1^	Neurogenic	Laparoscopic total colectomy, and ileoproctostomy
37	Female	50–55	15	Abnormal	Mild myogenic	Laparoscopic subtotal colectomy, and lateral side-to-side anastomosis of ascending colon and rectum
38	Female	50–55	14	Normal	Abnormal	Laparoscopic subtotal colectomy, and lateral side-to-side anastomosis of ascending colon and rectum
39	Female	60–65	7	Pathologic	Moderate mixed	Laparoscopic subtotal colectomy, lateral side-to-side anastomosis of ascending colon and rectum, ileostomy, complex enterolysis, and catheter drainage
40	Male	60–65	13	Abnormal (STC)	Abnormal	Laparoscopic subtotal colectomy, and lateral side-to-side anastomosis of ascending colon and rectum
41	Female	30–35	7	Pathologic	Mild mixed^2^	Laparoscopic total colectomy, ileoproctostomy, ileostomy, and complex adhesiolysis
42	Female	25–30	10	Abnormal (STC)	Mild myogenic	Laparoscopic total colectomy, and prophylactic ileostomy
43	Female	30–35	21	Abnormal (STC)	Severe myogenic	Laparoscopic total colectomy, and prophylactic ileostomy
44	Female	65–70	13	Abnormal (STC)	Neurogenic	Laparoscopic total colectomy, and ileoproctostomy, ileostomy, complex intestinal mucosa lysis
45	Female	60–65	7	Abnormal (STC)	Myogenic CST in left side and normal in right side	Laparoscopic subtotal colectomy, and ileoproctostomy, complex enterolysis
46	Female	50–55	19	Abnormal (STC)	Mixed^2^ (myogenic and neurogenic)	Laparoscopic total colectomy, and ileoproctostomy
47	Female	70–75	12	Abnormal (STC)	Mixed^2^ (moderate myogenic CST with neurogenic CST)	Laparoscopic total colectomy, and ileoproctostomy
48	Female	45–50	12	Normal	Moderate myogenic	Laparoscopic subtotal colectomy, and lateral side-to-side anastomosis of ascending colon and rectum
49	Male	55–60	14	Abnormal (STC)	Mixed^2^ (moderate myogenic CST with neurogenic CST)	Laparoscopic total colectomy, ileoproctostomy,complex adhesiolysis, and peritoneal drainage
50	Female	35–40	7	Abnormal (STC)	Mixed^2^ (severe myogenic CST in left side and neurogenic CST in right side)	Laparoscopic subtotal colectomy, lateral side-to-side anastomosis of ascending colon and rectum, and preventative fistulization
51	Female	45–50	14	Abnormal (STC)	Mixed^2^ (severe myogenic CST in right side with neurogenic CST in left side)	Laparoscopic subtotal colectomy, lateral side-to-side anastomosis of ascending colon and rectum, prophylactic ileostomy, complex adhesiolysis, and peritoneal drainage
52	Male	35–40	13	Normal	Mild myogenic	Laparoscopic total colectomy, and ileoproctostomy
53	Female	55–60	12	Abnormal (STC)	Moderate myogenic	Laparoscopic total colectomy, ileoproctostomy, and complex adhesiolysis
54	Female	40–45	22	Normal	Mixed^2^ (moderate myogenic CST with neurogenic CST)	Laparoscopic total colectomy, ileoproctostomy, transanorectal mucosa circumferential resection, and double luminal enterostomy
55	Female	60–65	12	Abnormal (STC)	Mixed^2^ (severe myogenic CST with neurogenic CST)	Laparoscopic subtotal colectomy, and lateral side-to-side anastomosis of ascending colon and rectum
56	Female	45–50	13	Abnormal (STC)	Mixed^2^ (moderate myogenic CST and partial neurogenic CST)	Laparoscopic total colectomy, and ileoproctostomy
57	Female	60–65	12	Abnormal (STC)	Moderate myogenic	Laparoscopic total colectomy, ileoproctostomy, complex adhesiolysis, and peritoneal drainage
58	Female	50–55	10	Abnormal (STC)	Moderate myogenic	Laparoscopic total colectomy, ileoproctostomy, complex adhesiolysis, and peritoneal drainage
59	Male	65–70	4	Mixed^1^	Severe myogenic	Laparoscopic total colectomy, ileoproctostomy, and temporary ileostomy, enterolysis
60	Male	60–65	13	Abnormal (STC)	Mixed^2^ (mild myogenic CST in right side and neurogenic CST in left side)	Laparoscopic total colectomy, ileoproctostomy, complex adhesiolysis, and peritoneal drainage
61	Female	55–60	10	Normal	Mild myogenic	Tissue-selecting therapy stapler
62	Female	40–45	9	Normal	Myogenic	Tissue-selecting therapy stapler
63	Female	25–30	10	Normal	Mild myogenic	Tissue-selecting therapy stapler
64	Female	70–75	7	Normal	Normal	Tissue-selecting therapy stapler
65	Female	45–50	6	Normal	Normal	Tissue-selecting therapy stapler
66	Female	45–50	16	Normal	Normal	Tissue-selecting therapy stapler
67	Female	45–50	11	Normal	Myogenic	Transvaginal repair of the rectal prolapse
68	Female	45–50	14	Normal	Mixed^2^	Tissue-selecting therapy stapler
69	Female	65–70	11	Normal	Normal	Tissue-selecting therapy stapler
70	Female	35–40	9	Normal	Normal	Tissue-selecting therapy stapler
71	Female	70–75	11	Normal	Normal	Tissue-selecting therapy stapler
72	Female	50–55	14	Normal	Normal	Partial rectal mucosa resection
73	Female	55–60	9	Normal	Myogenic	Tissue-selecting therapy stapler
74	Female	50–55	6	Mixed^1^	Normal	Posterior pelvic floor reconstruction, and tissue-selecting therapy stapler
75	Female	55–60	14	Abnormal (STC)	Normal	Tissue-selecting therapy stapler, and haemorrhoidectomy
76	Female	55–60	16	Normal	Normal	Gastric polyp electrocision
77	Male	65–70	14	Normal	Mixed (myogenic CST in left side and neurogenic CST in right side)	Laparoscopic terminal ileum single cavity fistula
78	Female	60–65	8	Normal	Normal	Procedure for prolapse and hemorrhoids, and rectocele repair, and haemorrhoidectomy
79	Female	55–60	8	Normal	Normal	Transanorectal mucous circumferential resection

Mixed^1^, mixed type of FC by CTT (combination of pathological and defecation disorder)

Mixed^2^, mixed type (myogenic and neurogenic type) of FC by HRCM

**Fig. 3:**
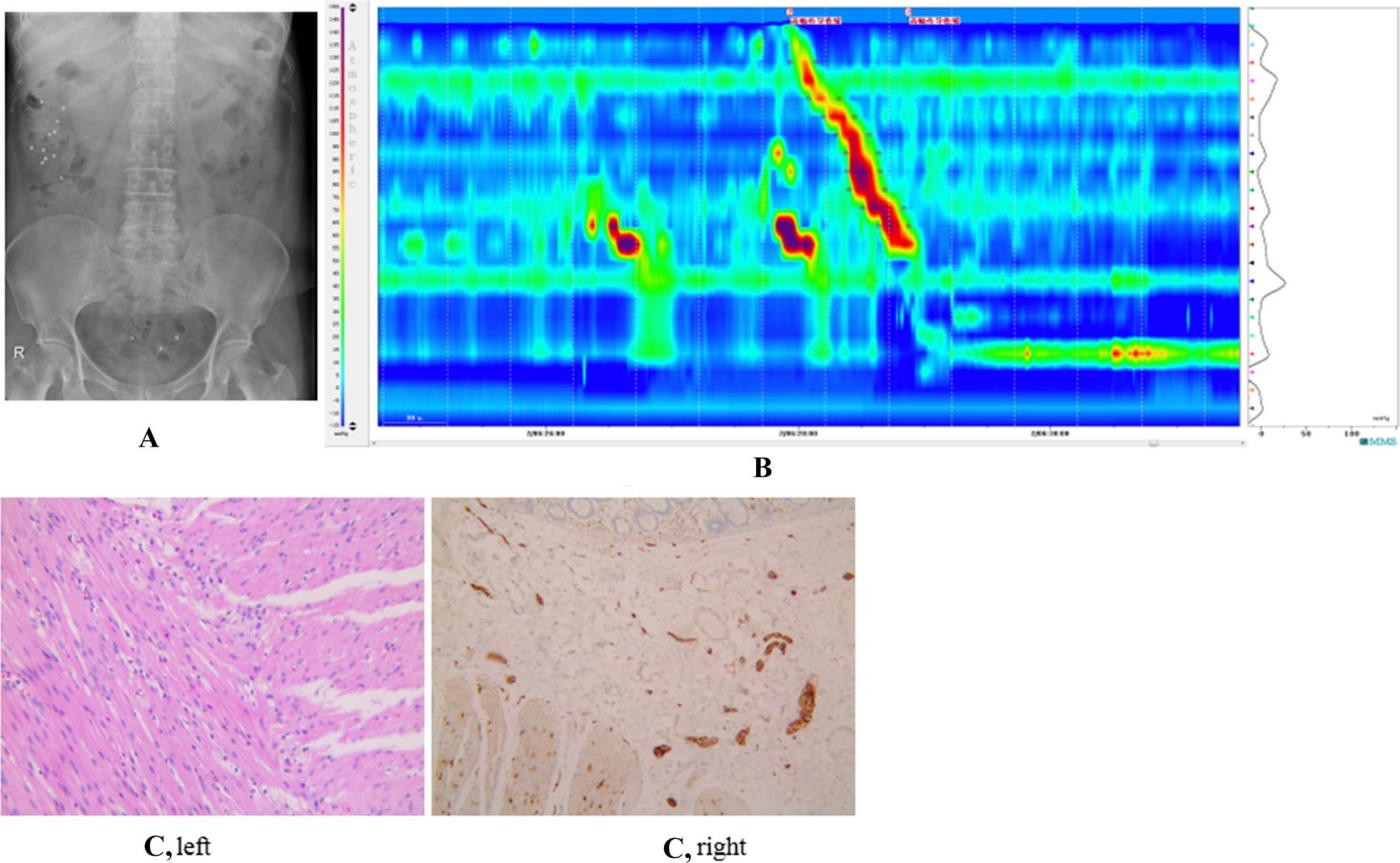
Both CTT and HRCM normal (negative) but HE staining positive images in a representative FC patient (Case 15). **A** CTT normal in Case 15. **B** HRCM normal in Case 15. **C** HE staining showed constipation in Case 15. Left, the intermuscular nerve was morphological abnormal and the nerve plexus was atrophied. Right, local hyperplasia was found in submucosal Meissener nerve plexus and Helen sheath nerve

**Fig. 4:**
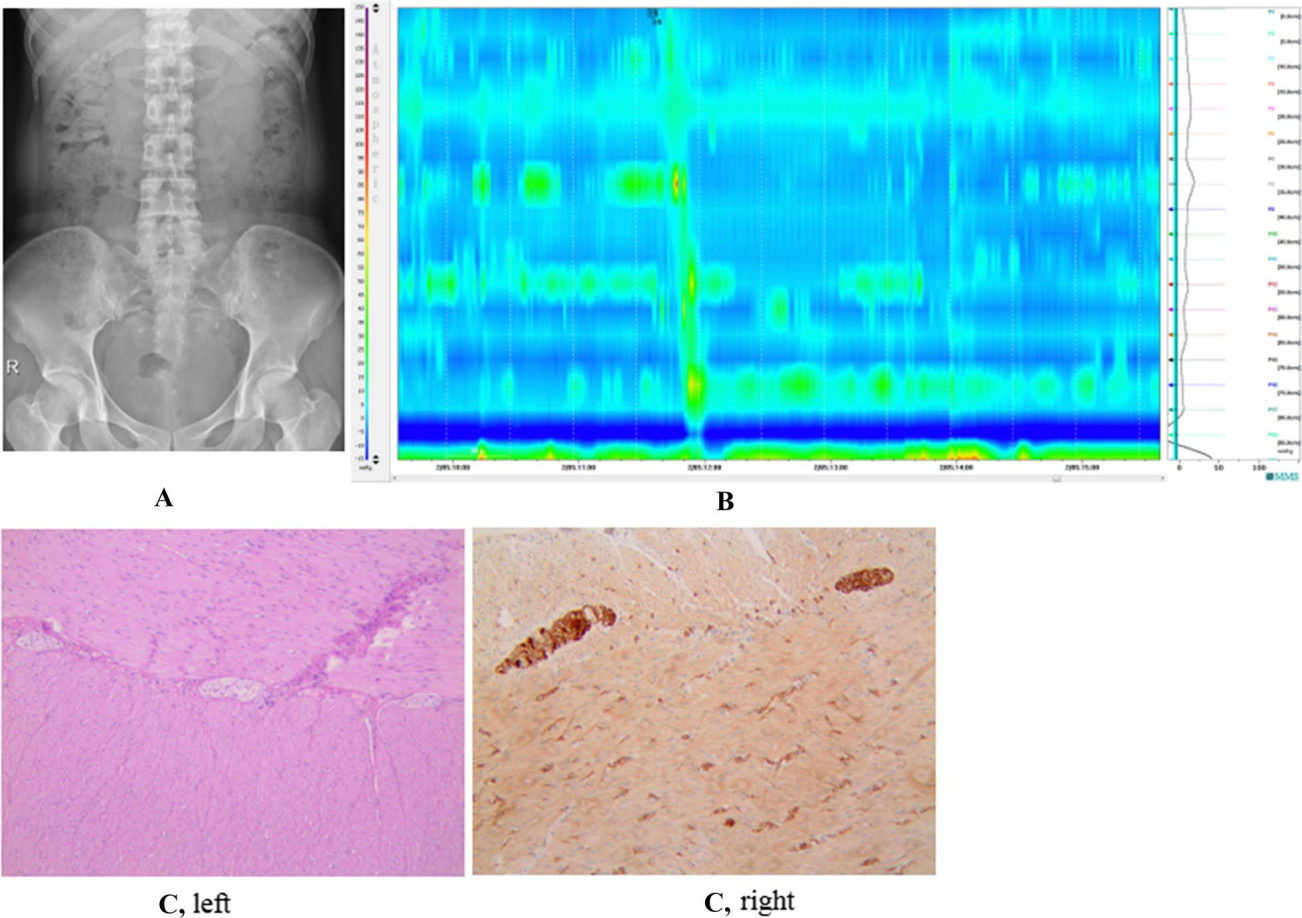
CTT normal (negative) but HRCM positive images in a representative FC patient (Case 54). **A** CTT normal in Case 54. **B** HRCM positive in Case 54. **C** HE staining showed constipation in Case 54. Left, the intermuscular nerve with abnormal morphology was unevenly distributed, and no obvious ganglion and smooth muscle cell edema were observed. Right, nonspecific esterase staining (NSE) showed no neurons in one ganglion

**Fig. 5:**
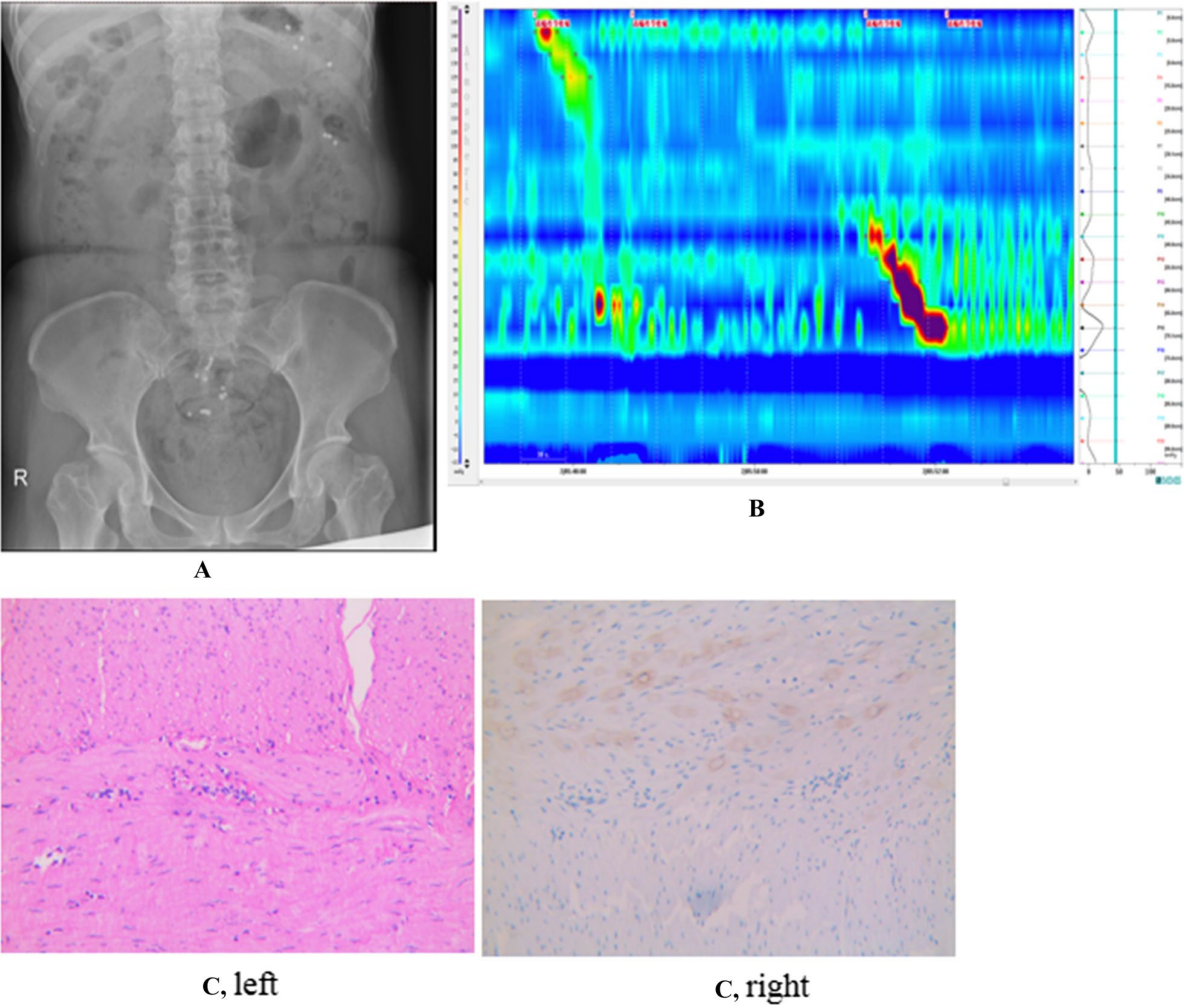
CTT positive but HRCM normal (negative) images in a representative FC patient (Case 33). **A** CTT positive in Case 33. **B** HRCM normal in Case 33. **C** HE staining showed constipation in Case 33. Left, intermuscular Auerbach nerve plexus atrophy was observed, with only a few atrophied nuclei. Right, nonspecific esterase staining (NSE) showed no neurons in the two Auerbach nerve plexuses

Among the 79 cases, 12 (#15/21/64/65/66/69–72/76/78/79, 12/79) with apparent symptoms of constipation were normal by both CTT and HRCM, while were confirmed to have STC (mild positive) by histological observation after surgical treatment due to unsatisfactory efficacy of conservative treatment. As shown in Fig. 3, in a representative case of FC patient (Case 15) both CTT (Fig. 3A) and HRCM were normal (negative, Fig. 3B) but the HE staining examination showed positive (Fig. 3C). For CTT examination, 12 cases (#22/38/48/52/54/61-63/67/68/73/77, 12/79) showed normal after CTT examination but abnormal after HRCM. A representative case (Case 54) was presented, showing CTT normal (negative, Fig. 4A) but HRCM positive (Fig. 4B), which was validated by HE staining examination (Fig. 4C). In contrast, 4 cases (#33/65/74/75, 4/79) showed normal after HRCM but abnormal after CTT. A representative case (Case 33) with normal HRCM (Fig. 5A) but positive CTT (Fig. 5B) results was similarly presented, as evidenced by HE examination (Fig. 5C).

CTT can only determine normal, and pathological and mixed (combination of pathological and defecation disorder) constipation (Table 1). In contrast, HRCM can identify the detailed types of STC, such as myogenic colonic slow transmission (CST, #1–7, 10, 13, 14, 16–18, 22, 23, 24, 26, 29, 30, 34, 35, 62, 67, and 73), partial myogenic CST (#8, 20, 31, and 45, e.g. in Case 45 left side showed myogenic CST but right side normal), mild myogenic CST (#27, 32, 37, 42, 52, 61, and 63), moderate myogenic CST (#48, 53, 57, and 58), severe myogenic CST (#43, and 59), neurogenic CST (#25, 36, and 44), partial neurogenic CST (#19), mixed CST (i.e. combination of myogenic and neurogenic CST, #9, 11, 12, 28, 46, 68, and 77). Mixed CST comprised several subtypes, including mild myogenic CST in right and neurogenic CST in left side (#60), myogenic CST in left and neurogenic CST in right side (#77), moderate myogenic CST and partial neurogenic CST (#56), mild mixed CST (#41), moderate mixed CST (#39), severe myogenic CST with neurogenic CST (#55), and moderate myogenic CST with neurogenic CST (#47, 49, and 54). HRCM even could determine the sites of different kinds of CST, e.g. severe myogenic CST in left side with neurogenic CST in right side (#50), and severe myogenic CST in right side with neurogenic CST in left side (#51).

## Discussion

It is vital important for clinicians to determine and classify constipation and to accurately assess the clinical stages and identify the lesions in the colon segments to select the appropriate treatments. The current recommended method by the related guideline is the CTT to assess the general colonic peristalsis function, but it cannot further classify constipation and accurately identify the diseased colonic segments, let alone determination of the colonic peristaltic functions. HRCM has been increasingly developing for more effectively evaluating the colonic peristaltic contractions of the entire human colons, such as colon motility, manometry, operation, and colonic and anal motor activity [8–13], in the diagnosis and classification of constipation. Until now, however, it is still not clear if HRCM is superior to and can substitute CTT in the diagnosis of constipation.

In this study, we reviewed the clinical information of 79 patients with FC who underwent the CTT and whole HRCM in sequence before colonectomy to compare the diagnostic efficacy of HRCM vs CTT. We showed that the diagnostic accuracy of HRCM for STC was higher than that of CTT (81.0% vs 69.6%), and the false negative ratio of HRCM was lower (19.0% vs 30.4%). In addition, 12 cases showed normal by CTT examination but abnormal by HRCM, while only 4 cases showed normal by HRCM but abnormal by CTT. This indicates the advantage of HRCM in the diagnosis of FC in comparison with CTT.

We reported that HRCM could identify the detailed types of STC, such as myogenic, partial myogenic, mild myogenic, moderate myogenic, severe myogenic, neurogenic, partial neurogenic, and mixed STC. Moreover, mixed STC comprised several subtypes, including mild myogenic STC in right and neurogenic STC in left side, myogenic STC in left and neurogenic STC in right side, moderate myogenic STC and partial neurogenic STC, mild mixed STC, moderate mixed STC, severe myogenic STC with neurogenic STC, and moderate myogenic STC with neurogenic STC. We showed HRCM even could determine the sites of the various kinds of STC, e.g. severe myogenic STC in left side and neurogenic STC in right side, and severe myogenic STC in right side and neurogenic STC in left side. Such detailed observation on FC by HRCM has not been previously reported.

Worth of mention, among the 79 cases, 12 (#15, 21, 64-66, 69-72, 76, 78, and 79, 12/79) with apparent symptoms of constipation were normal by both CTT and HRCM, while they were all confirmed STC (mild positive) by histological observation. This indicates that both CTT and HRCM have defaults (false negative) and not sufficient in the diagnosis of FC because colonic peristalsis based on which CTT and HRCM determine constipation may be influenced by various factors (such as nervousness and diets) during the detection period, thus resulting in false negative result. Therefore, CTT and HRCM detection results should be combined with clinical symptoms in the diagnosis of constipation, and combined application of CTT and HRCM and following validation by histological observation are needed.

There were limitations in this study. This was a retrospective study that there would be some bias in selection of cases since only patients who underwent both HRCM and CTT and received surgery were reviewed. In addition, the case number was not big. Next, prospective studies involving more patients with HRCM or CTT examination, or the combination will be carried out to validate this study.

## Conclusion

In summary, we concluded that: 1) HRCM is more effective in the diagnosis of FC than CTT, both in the diagnostic accuracy and in obtaining adequate information of FC, 2) CTT is indispensable because it increases the diagnostic value of HRCM, 3) both CTT and HRCM are not sufficient enough in the diagnosis of FC, which calls validation of histological observation. Next, more patients will be recruited to validate this result, and the diagnostic value of HRCM in the identification of the subtypes of FC will be assessed as well.

## Data Availability

The datasets used and/or analyzed during the current study available from the corresponding author on reasonable request.

## Abbreviations

STC: Slow-transit constipation
FC: Functional constipation
CTT: Colonic transit test
HRCM: High-resolution colonic manometry
LAPC: Low amplitude peristaltic contraction
HE: Hematoxylin and eosin
CST: Colonic slow transmission
